# Monocyte-derived dendritic cells from HLA-B27^+^ axial spondyloarthritis (SpA) patients display altered functional capacity and deregulated gene expression

**DOI:** 10.1186/s13075-014-0417-0

**Published:** 2014-08-21

**Authors:** Alice Talpin, Félicie Costantino, Nelly Bonilla, Ariane Leboime, Franck Letourneur, Sébastien Jacques, Florent Dumont, Sonia Amraoui, Charles-Antoine Dutertre, Henri-Jean Garchon, Maxime Breban, Gilles Chiocchia

**Affiliations:** INSERM U987, Laboratoire d’excellence INFLAMEX, Université Versailles-Saint-Quentin, Versailles, 78000 France; UFR des Sciences de la Santé, Simone Veil, Versailles Saint Quentin en Yvelines Université, Montigny-Le-Bretonneux, 78180 France; Rheumatology Division, Ambroise Paré Hospital (AP-HP), Boulogne-Billancourt, 92100 France; Institut Cochin, INSERM U1016, CNRS (UMR 8104), Université Paris-Descartes, Sorbonne Paris-Cité, 75014 France; Antigen Presentation by Dendritic Cell Team; Institut Cochin, INSERM U1016, CNRS (UMR 8104), Université Paris-Descartes, Sorbonne Paris-Cité, 75014 France; Duke-NUS Graduate Medical School, Program in Emerging Infectious Disease, Singapore, Singapore; Genetics Division, Ambroise Paré Hospital (AP-HP), Boulogne-Billancourt, 92100 France

## Abstract

**Introduction:**

This study aimed to compare the functional capacity and gene expression profile of monocyte-derived dendritic cells (MD-DCs) in HLA-B27^+^ axial spondyloarthritis (SpA) patients and healthy controls.

**Methods:**

MD-DCs were differentiated with interleukin 4 (IL-4) and granulocyte-macrophage colony-stimulating factor (GM-CSF) for seven days, starting from purified CD14^+^ monocytes and stimulated with lipopolysaccharide (LPS) for six and twenty four hours. Their capacity to stimulate allogeneic CD4^+^ T cells from unrelated healthy donor was tested. Transcriptomic study was performed with Affymetrix HuGene 1.0 ST microarrays. Gene expression levels were compared between patients and controls using a multivariate design under a linear model (LIMMA). Real-time quantitative PCR (qRT-PCR) was performed for validation of the most striking gene expression differences.

**Results:**

The stimulatory capacity of allogeneic CD4^+^ T cells by MD-DCs from SpA patients was decreased. Transcriptomic analysis revealed 81 genes differentially expressed in MD-DCs between SpA patients and controls (*P* <0.01 and fold-change <0.66 or >1.5). Four selected genes were validated by qRT-PCR: *ADAMTS15, CITED2, F13A1* and *SELL*. Expression levels of *ADAMTS15* and *CITED2,* encoding a metallopeptidase and a transcription factor, respectively, were inversely correlated with each other (R = 0.75, *P* = 0.0003). Furthermore, *in silico* analysis identified several genes of the Wnt signaling pathway having expression co-regulated with *CITED2*.

**Conclusion:**

This study revealed altered function and gene expression pattern in MD-DCs from HLA-B27^+^ axial SpA. Co-expression study showed an inverse correlation between *ADAMTS15* and *CITED2*. Moreover, the Wnt signaling pathway appeared as deregulated in SpA MD-DCs, a finding which may be connected to Th17-driven inflammatory responses.

**Electronic supplementary material:**

The online version of this article (doi:10.1186/s13075-014-0417-0) contains supplementary material, which is available to authorized users.

## Introduction

Spondyloarthritis (SpA) is a chronic inflammatory rheumatic disorder, with a prevalence of around 0.42% in Caucasian populations [1]. Joint inflammation is responsible for pain and stiffness but long-term outcome is mainly determined by new bone formation, which can lead to complete ankylosis. Effective treatments such as TNF blockers can temporarily suppress inflammation, but none has yet proven to affect long-term disease outcome. Therefore, there is a need to better understand pathological mechanisms controlling both initiation and progression of SpA.

Genome-wide gene expression analysis is a powerful approach to identify molecular mechanisms responsible for a disease. Microarray studies have already been conducted in SpA, most of them focusing on the ankylosing spondylitis (AS) subtype [2–7]. Each of those studies identified several genes differentially expressed between patients and controls but there was very little overlap between their results, maybe due to the variety of protocols.

Several studies support an important role of dendritic cells (DCs) in the pathogenesis of SpA. Indeed, aberrant functions of DCs have been demonstrated in an HLA-B27/human β2-microglobulin transgenic rat model of SpA [8]. First, DCs from these rats have a decreased capacity to stimulate primary allogeneic or syngeneic T cell responses [9]. Furthermore, the proportion of conjugates formed between HLA-B27 DCs and naïve CD4^+^ T cells is reduced. Moreover, mature HLA-B27 molecules expressed by DCs appear to impair the formation of an antigen-independent immunologic synapse with naïve CD4^+^ T cells by interfering with the engagement of co-stimulatory molecules [10]. Finally, B27 transgenic rat DCs were shown to favor Th17 expansion [11,12] and to alter regulatory T cell function, resulting in decreased IL-10 and enhanced IL-17 production [13].

On this basis, we hypothesized that DCs also play an important role in human disease, as proposed in the HLA-B27 transgenic rat model. The aim of the current study was to compare functional capacity and the gene expression profile of monocyte-derived DCs (MD-DCs) in patients with HLA-B27^+^ axial SpA and healthy controls.

## Methods

### Patients and controls

We studied three different groups of patients and controls: 10 patients and 5 controls for monocyte subset characterization by flow cytometry, 19 patients and 24 controls for mixed lymphocyte reaction and 9 patients and 10 controls for the MD-DC transcriptomic study (6 patients and 4 controls were investigated for the two latter). All the patients were HLA-B27^+^ and fulfilled the Assessment of SpondyloArthritis International Society classification criteria for axial SpA [14]. Controls were healthy blood donors.

Patients’ and healthy controls’ characteristics are summarized in the Additional files (see Additional file 1: Table S1 and Additional file 2: Table S2, respectively). All participants in the study gave written informed consent and the study was approved by local ethics committee of Ile-de-France XI (Saint-Germaine-en-Laye France).

### Cell isolation, culture and stimulation

Peripheral blood mononuclear cells (PBMCs) were isolated from 50 mL of blood by gradient separation on Ficoll density gradient centrifugation (STEMCELL Technologies, Grenoble, France). Monocytes used to generate MD-DCs were purified by magnetic cell sorting using anti-CD14 monoclonal antibody (mAb)-coated beads (BD IMag, Le Pont de Claix, France). Sorted monocytes were morphologically homogeneous with 99% of CD14^+^ cells, as determined by flow cytometry.

Monocytes were further cultured for 6 days in 24-well plates (400,000 cells/500 μL) in Roswell Park Memorial Institute (RPMI) 1640 medium supplemented with 10% heat-inactivated fetal calf serum, 100 U/mL penicillin, 100 μg/mL streptomycin, 500 U/mL recombinant human granulocyte-macrophage colony stimulating factor (rhGM-CSF) and 500 U/mL rhIL-4 (AbCys, Paris, France). Then, the MD-DCs were stimulated or not with lipopolysaccharide (LPS) from *Escherichia coli* (LPS, Sigma-Aldrich, St Louis, MO, USA) at a concentration of 100 ng/mL for the last 6 or 24 hours of culture (further referred to as time points H0, H6 and H24).

CD4^+^ T cells were purified from PBMCs from two unrelated healthy donors by magnetic cell sorting using anti-CD4 monoclonal antibody (mAb)-coated beads (BD IMag), and stored frozen until used for mixed lymphocyte reaction (MLR).

### Flow cytometry

To characterize monocyte subsets, freshly purified PBMCs were analyzed by six-color flow cytometry on FACS LSRII apparatus. The gating strategy was based on a previous report [15]. Monocytes were subdivided into three major subsets: classical CD14^++^CD16^−^, intermediate CD14^++^CD16^+^ and non-classical CD14^+^CD16^++^ monocytes. The following anti-human mAbs were used: CD45-Amcyan (BD Biosciences), HLA-DR-PerCP (BD Biosciences), CD19-ECD (Beckman Coulter), CD14-QDot655 (Invitrogen), CD16-APC-H7 (Beckman Coulter, Villepinte, France). The Live/Dead blue Dye (Invitrogen) was used to exclude dead cells.

Samples of the purified monocytes used to generate MD-DCs and of the resulting MD-DCs were routinely stained with the following anti-human mAbs: CD14-FITC, CD11c-APC, CD40-PE, HLA-I-FITC, HLA-DR-PerCP, CD80-PE, CD83-APC and CD86-FITC (all from BD Bioscience) and analyzed by flow cytometry on FACS canto II apparatus (BD Biosciences).

### Mixed lymphocyte reaction (MLR)

Purified allogeneic CD4^+^ T cells (10^5^ cells per well) from healthy donors were cultured with unstimulated (H0) or LPS-stimulated (H6, H24) MD-DCs (10^4^ cells per well), in 96-well flat-bottomed culture dishes in a final volume of 200 μL. Proliferation of T cells was assayed by measuring incorporation of ^3^H-deoxythymidine added (0.5 μCi per well) after 6 days of culture, using a Microbeta scintillation counter (Wallac, Turku, Finland). Data are expressed as the mean counts per minute (CPM) in triplicate wells. An MLR index (ratio of CPM of MLR on CPM of CD4^+^ T cells only) was used to represent CD4^+^ T cell proliferation. Two stored CD4^+^ T cell batches from different healthy donors were sequentially used for MLR in two sets of experiments, each including equivalent numbers of patient and control MD-DC samples. As there was no statistically significant difference in the results between both sets of experiments, we pooled them. The Wilcoxon test was used to compare MLR indices between patients and controls at each stimulation time point.

### Transcriptomic study

#### RNA isolation

MD-DCs were disrupted and homogenized using RLT buffer (Qiagen, Valencia, CA, USA). Total RNA was isolated using RNeasy Mini Kit (Qiagen). RNA quantity and quality were assessed using Agilent 2100 Bioanalyzer (Agilent, Santa Clara, CA, USA). Only samples with an RNA integrity number (RIN) above 8 were further processed.

#### Microarray hybridization

RNA was reverse-transcribed, converted to biotinylated complementary RNA using standard Affymetrix protocol (Affymetrix, Santa Clara, CA, USA) and hybridized to the Affymetrix GeneChip Human Gene 1.0 ST Array by the genomic platform of the Cochin Institute.

#### Differential gene expression validation by qRT-PCR

For validation, the relative gene expression levels of candidate genes identified through the foregoing microarray study were further quantified using qRT-PCR. Briefly, RNA treated with DNase I (Invitrogen) was reverse-transcribed using SuperscriptII (Invitrogen) and then quantified using the SYBR green PCR Master Mix (Applied Biosystems) and the 7300 Real-Time PCR System (Applied Biosystems). Primers were purchased from Eurofins MWG (nucleotide sequences of the PCR primers are available in Additional file 3: Table S3). The experiment design included three technical replicates.

#### Statistical analysis

Raw Affymetrix data (.cel files) from 57 arrays (corresponding to H0, H6 and H24 time points of stimulation by LPS for 19 subjects) were transformed by the Robust Multichip Analysis (RMA) method using Bioconductor in R software (library Affy) [16]. This transformation included background correction, normalization and summarization of expression values using Brainarray version 15 custom chip definition (cdf) files generated with the Ensembl annotation set [17]. Before analysis, genes were filtered on annotation and expression level mean with a cut off at 6.11 (corresponding to the mean of expression level of both anti-genomic and intronic probes of housekeeping genes). Gene expression levels were then fitted to a bivariate linear model including disease status and time point after stimulation, using the LInear Models for Microarray data (LIMMA) package of Bioconductor in R environment to determine differentially expressed genes [18]. The threshold for differential gene expression between patients and controls was set to a global fold change above or below 1.5 with a nominal *P*-value below 0.01.

For qRT-PCR validation, gene expression data were computed with the ΔΔCq method, using three housekeeping genes: *RPL30*, *β-ACT* and *GAPDH*. Then, two-way analysis of variance (ANOVA) was used to test differential gene expression among samples from SpA and controls without (baseline; no treatment) and with LPS treatment for 6 and 24 hours. In case of statistical significance (*P* <0.05), post hoc *t*-tests with the Bonferroni correction allowed us to perform pairwise comparisons.

### Paired genes co-expression study and molecular pathway analysis

Pairwise correlations between gene expression levels were tested using the nonparametric Spearman test. We used the commercial software Genomatix Pathway System [19] to identify significantly enriched pathways and functional themes, among the list of the best correlated genes with genes of interest.

## Results

### Functional impairment of MD-DCs from SpA patients

To verify that MD-DCs were differentiated from comparable monocyte populations between SpA patients and controls, we analyzed monocytes subsets in both groups. As shown in an additional file, the distribution of the classical, intermediate and non-classical circulating subsets among CD45^+^ PBMCs was similar in patients and controls (see Additional file 4: Figure S1).

The cells obtained after 7 days of differentiation in culture in patients and controls were uniformly CD14^−^, CD11c^+^, CD40^+^, HLA-DR^+^, CD83^dim^ and CD86^+^, consistent with a MD-DC phenotype. Moreover, HLA-DR, CD80 and CD86 expression increased and CD83 was induced on MD-DCs after LPS stimulation for 24 hours (Additional file 5: Figure S2).

**Figure 1:**
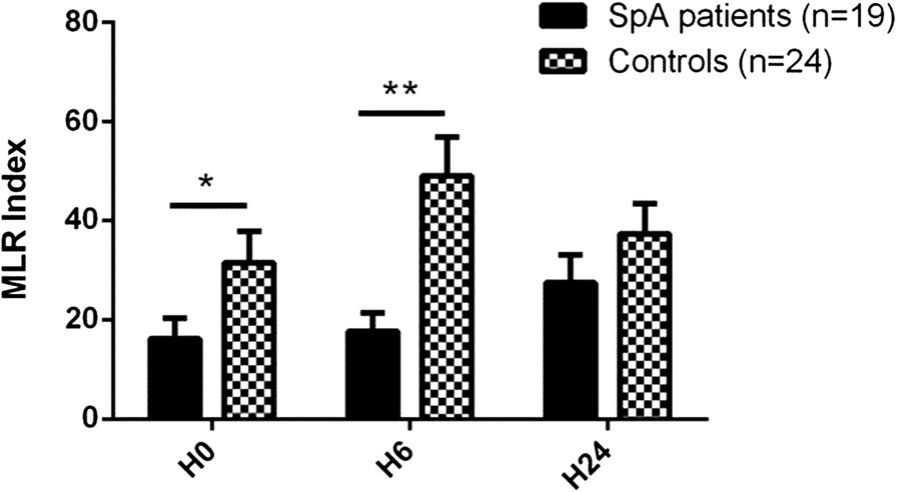
**Impaired allogeneic T cell stimulatory capacity of monocyte-derived dendritic cells (MD-DCs) from spondyloarthritis (SpA) patients.** MD-DCs from SpA patients and healthy controls that were left unstimulated at baseline (H0), or were stimulated with lipopolysaccharide (LPS) for 6 h or 24 h (H6 and H24, respectively) were tested for their capacity to stimulate *in vitro* allogeneic CD4^+^ T cells. Results are expressed as ^3^H-deoxythymidine incorporation after 6 days of mixed lymphocyte reaction, in counts per minute (CPM) ratio. Bars represent the mean proliferation index and standard error of the mean induced by MD-DCs from 19 SpA patients and 24 healthy controls. The Wilcoxon test showed significant differences at H0 (*P <0.05) and H6 (**P <0.01). MLR, mixed lymphocyte reaction.

In the rat model of SpA, HLA-B27 transgenic DCs have a decreased capacity to stimulate allogeneic T cells. Thus, we tested the capacity of MD-DCs from HLA-B27^+^ SpA patients to prime allogeneic CD4^+^ T cells using an *in vitro* proliferation assay. A weaker proliferation of CD4^+^ T cells was observed with SpA MD-DCs as compared to controls, which was statistically significant before and after 6 hours of LPS exposure (*P* <0.05 and *P* <0.01, respectively; Figure 1).

### Identification of differentially expressed genes in MD-DCs between patients and controls

To investigate the mechanisms underlying the functional defect of MD-DCs from SpA patients, we then compared gene expression levels in those cells between SpA and healthy donors upon stimulation with LPS. After filtering on gene expression level and gene annotation, 13,021 genes (57.3% of the genes present in the microarray) were kept for further analysis. Expression data for MD-DCs either left unstimulated or stimulated with LPS for 6 or 24 hours were first globally compared between SpA patients and healthy subjects. Unsupervised hierarchical clustering showed that samples were perfectly grouped according to LPS stimulation time points (Additional file 6: Figure S3) but not to the disease status. Linear modeling nevertheless identified 81 genes differentially expressed between patients and controls at any time point, with a nominal *P*-value <0.01 and a fold change below 0.66 or greater than 1.5 (Additional file 7: Table S4). Of this set of genes, 61 were downregulated and 20 were upregulated in patients.

**Figure 2:**
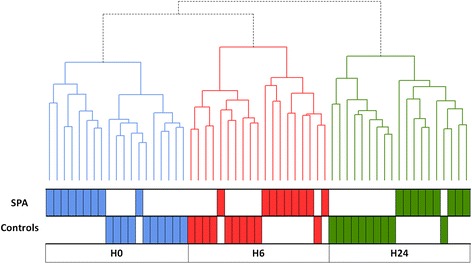
**Unsupervised hierarchical clustering of 57 samples based on microarray expression levels of the 81 differentially expressed genes between spondyloarthritis (SpA) patients and controls.** Each time point is color-coded (baseline (H0): blue, 6 h (H6): red, 24 h (H24): green). Samples are clustered on the horizontal axis (top row: SpA patients, bottom row: healthy controls), with the length on the vertical axis representing the degree of correlation between samples.

Using the extraction of the dataset corresponding to these 81 genes, LPS stimulation time points were perfectly separated by unsupervised hierarchical clustering. Furthermore, patients and controls were now clearly discriminated, with only four misclassifications (one at H0, two at H6 and one at H24) (Figure 2).

**Figure 3:**
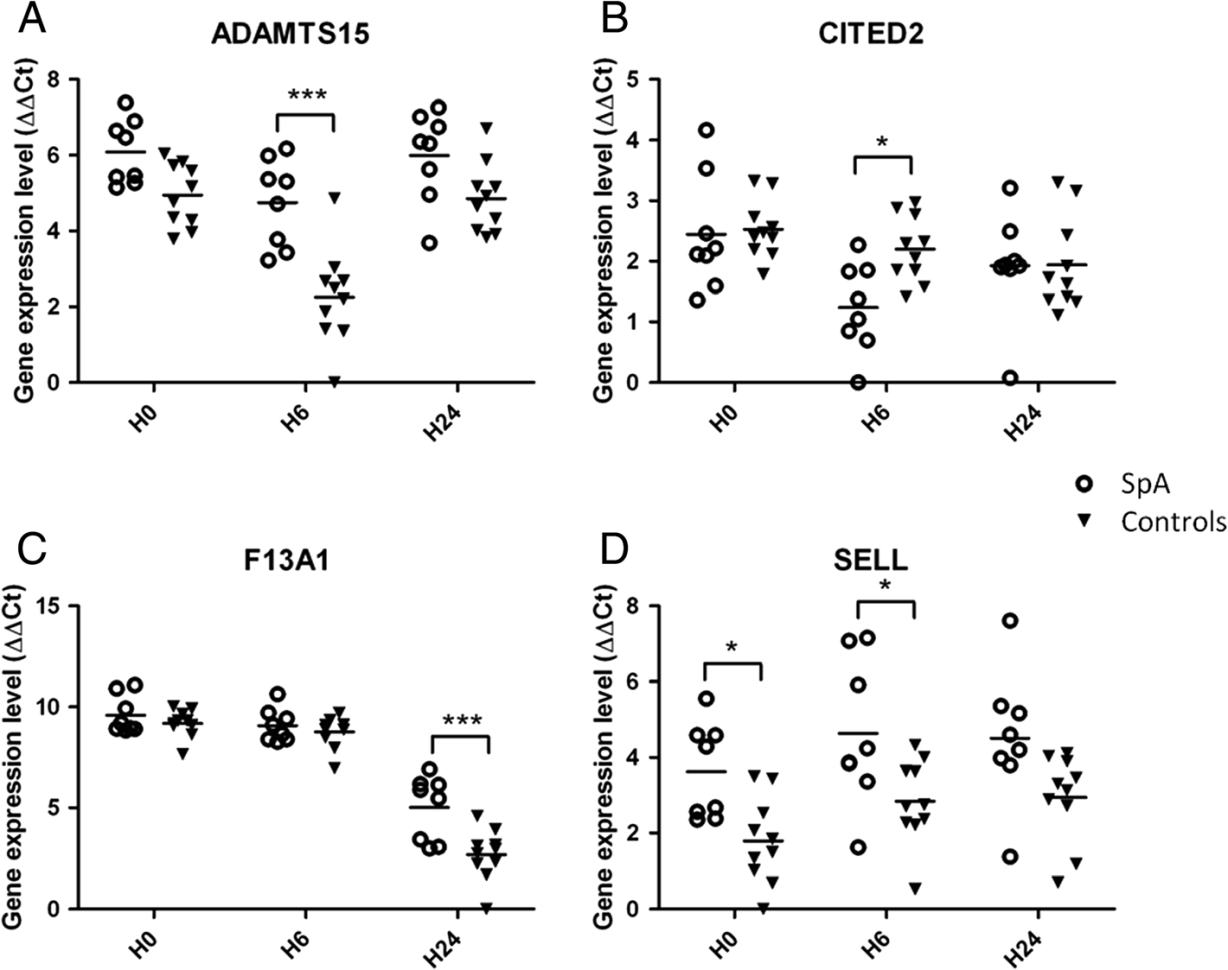
**Scatter plots showing qRT-PCR expression levels in spondyloarthritis (SpA) and controls of the 4 selected genes:** ***ADAMTS15*** **(A),** ***CITED2*** **(B),** ***F13A1*** **(C) and** ***SELL*** **(D).** The x-axis of the plots represents the three lipopolysaccharide-stimulation time points (baseline (H0), 6 h (H6) and 24 h (H24)) and the y-axis shows the log2 of gene expression level normalized with housekeeping genes (∆∆Ct). *P <0.05, ***P <0.0005, for SpA versus controls.

Four candidate genes were chosen for validation study on the basis of their *P*-value, fold change and biological relevance: *ADAMTS15, CITED2, F13A1* and *SELL*. For all four genes, the qRT-PCR data produced with the RNA samples used for the microarray study confirmed the significant differences in expression and the direction of changes between SpA patients and controls (Table 1, Figure 3): *ADAMTS15*, *F13A1* and *SELL* were significantly upregulated in SpA samples, whereas *CITED2* was downregulated.

**Table 1:** **qRT-PCR expression analysis of four selected genes in monocyte-derived dendritic cells from spondyloarthritis patients, as compared to controls**

**Gene**	**Microarray**	**qRT-PCR**				
	***P-*** **value**	***P*** **-value**		**Fold change**		
		**Disease**	**Disease/time**	**H0**	**H6**	**H24**
***ADAMTS15***	0.0003	**0.0005**	**0.04**	2.2	**5.63**	2.2
***CITED2***	0.0007	0.21	**0.031**	0.94	**0.51**	0.99
***F13A1***	0.00006	**0.019**	**0.001**	1.32	1.25	**5.05**
***SELL***	0.006	**0.012**	0.8	**3.57**	**3.47**	2.97

*P*-value is reported considering the global effect, the effect of the disease status only or the effect of the disease status adjusted for time. Bold text indicates significant *P*-values and at what time the modulation of gene expression was significantly different between patients and controls.

### Co-regulated genes in SpA patients

**Figure 4:**
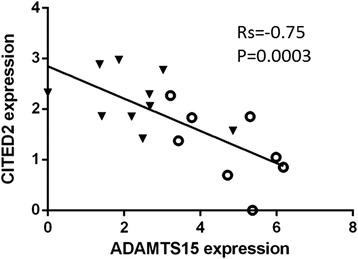
**Correlation of qRT-PCR expression levels between** ***CITED2*** **and** ***ADAMTS15.*** Expression is shown for H6 time-point in mixed spondyloarthritis (open circles) and control (black triangles) samples. *P*-value (P) and nonparametric Spearman correlation coefficient value (rho) are shown.

Examination of gene expression data indicated that some of them displayed similarity in their expression profile, suggesting co-regulation. Indeed, we found a highly significant negative correlation between *ADAMTS15* and *CITED2* expression levels at H6 in both patient and control groups (combined data: rho = −0.75; *P* = 0.0003; Figure 4).

**Figure 5:**
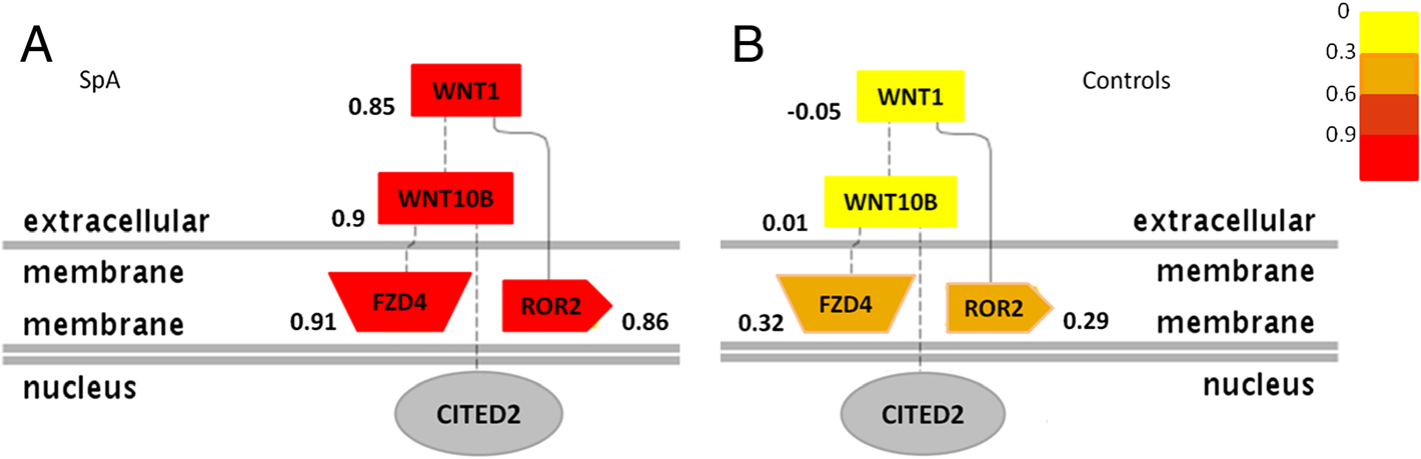
**Co-expression of the Wnt signaling pathway with** ***CITED2*** **in spondyloarthritis (SpA).** Graphical view of the Wnt pathway identified by studying genes co-expressed at 6 h (H6) with *CITED2* in the whole microarray dataset in patients **(A)** and in controls **(B)**. Numbers and color code (scale on the right side) indicate nonparametric Spearman correlation coefficient of the network's gene with CITED2.

Considering the transcriptional co-activator function of *CITED2* and its downregulation in SpA MD-DCs, we sought whether genes other than *ADAMTS15* shared a similarly co-regulated expression pattern in patient MD-DCs. We tested the correlation of the genome-wide expression matrix of patient MD-DCs with *CITED2* expression in a pairwise fashion. A set of 222 genes was found significantly correlated with *CITED2* (*P* <0.01). They were analyzed with the Genomatix web platform in order to interrogate their biological relevance. This revealed a significant enrichment of genes belonging to the Wnt signaling pathway (*P* = 2.48 × 10^-4^) in the SpA group. In the controls, there was no significant correlation between expression levels of *CITED2* and the four genes identified in the Wnt pathway (that is, *WNT1*, *WNT10B*, *FZD4* and *ROR2*) (Figure 5). This co-regulation pattern involving four genes of the Wnt signaling pathway was therefore specifically associated with the disease.

## Discussion

Starting from the hypothesis that aberrant DCs function could play a critical role in the development of SpA, we showed here for the first time that the capacity of MD-DCs to stimulate allogeneic CD4^+^ T cell response was impaired in SpA patients compared to controls. The genome-wide transcriptome of these MD-DCs elicited by LPS stimulation revealed 81 genes differentially expressed, 4 of which have been validated by RT-qPCR. Finally, we identified the Wnt signaling pathway as dysregulated in patients.

Our choice to study *in vitro-*differentiated DCs rather than DCs purified from peripheral blood had the advantage to remove those cells from *in vivo* influences, such as non-specific acute inflammation and drug therapy. Therefore, the differences that we observed between DCs from SpA and controls were more likely to be intrinsic to the DC and could be involved in the disease mechanism.

First, we demonstrated that MD-DCs differentiated from similar monocyte populations in both groups. Then, we observed that the ability of SpA MD-DCs to stimulate allogeneic CD4^+^ T cell proliferation was markedly defective, as compared to controls, as previously shown in SpA-prone HLA-B27 transgenic rats. The molecular basis for this impaired DC function has yet to be elucidated.

To investigate this impairment, we have studied the time-dependent global transcriptome of MD-DCs in response to LPS stimulation. A number of microarray-based studies have previously been undertaken in AS, on whole blood cells [3–5], PBMCs [6,7] or macrophages [2]. However, no transcriptomic analysis had been conducted on DCs. Of note, as we worked on a homogeneous cell population rather than on cell mixture, our results were not confounded by variations in the composition of the cell populations [20]. The time-dependency of our gene profiling is also a novel aspect, rarely investigated in humans.

Our analysis revealed 81 genes differentially expressed in resting and/or LPS-stimulated MD-DCs between SpA patients and controls. Of note, we did not identify a reverse interferon signature, such as in HLA-B27 transgenic rat splenic DCs [21] or in SpA patients monocyte-derived macrophages [2]. Such a discrepancy could be explained by differences in culture conditions. Here, we used IL-4 to differentiate MD-DCs, a cytokine that opposes interferon and interferon-induced gene expression and this might have blunted interferon-related differences between groups. Based on the magnitude of their variation between patients and controls, the level of statistical significance, and their biological relevance, we selected four of these genes that we validated using qRT-PCR.

We found an increased expression of *ADAMTS15* (A Disintegrin And Metalloproteinase with ThromboSpondin motif) in SpA. Although the ADAMTS family has not yet been implicated in this disorder, numerous studies have identified a role for metalloproteinases (MMPs) in SpA susceptibility and severity [22–24]. Of note, *ADAMTS15* was shown to be expressed in the joint with decreased expression in osteoarthritis [25]. Thus, ADAMTS15 could be implicated in the cartilage and/or bone turnover that takes place during joint inflammation, such as in SpA.

*CITED2* functions as a context-dependent transcriptional modulator to up- or downregulate the expression of specific genes [26–28]. Here, the expression of *CITED2* was downregulated in SpA MD-DCs. We further showed an inverse correlation between *CITED2* and *ADAMTS15* expression after 6 hours of LPS treatment. Interestingly, similar inverse correlation has previously been reported between *CITED2* and several MMP family members [29,30]. MMPs constitute a very important group of proteolytic enzymes in joint tissues. Thus, it has been suggested that CITED2 exerted chondroprotective effects through MMP downregulation [31]. The downregulation of *CITED2* and the inverse correlation between *CITED2* and *ADAMTS15* expression that we observed here suggest that DCs or other phagocytic mononuclear cells, such as macrophages and osteoclasts, could be implicated in SpA joint resorption through heightened metallopeptidase activity.

*In silico* pathway analysis conducted on *CITED2* co-expressed genes highlighted downregulation of several factors belonging to the canonical (that is, *WNT1*, *WNT10B* and *FRZL4*) and non-canonical (*ROR2*) Wnt signaling pathways, of potential relevance for SpA pathogenesis. First, factors of the canonical Wnt pathway were shown to play a crucial role either in bone formation or destruction in inflammatory arthritis [32,33], such as WNT10b [34,35]. On the other hand, activation of canonical Wnt-β-catenin signaling in DCs was shown to concur to regulatory T cell differentiation and conversely to inhibit Th17 differentiation [36]. Moreover, ROR2 is a signaling component of the non-canonical Wnt pathway acting on actin cytoskeleton to stimulate cell migration [37,38]. Interestingly, altered T cell stimulation was linked to defective cytoskeleton dynamics in HLA-B27 transgenic rat DCs [39].

*F13A1* encodes the coagulation factor XIII A subunit, a transglutaminase enzyme. It has multiple extra- and intracellular functions, including a role in cartilage and bone development. Hence, increased factor XIII A expression has previously been associated with cartilage ageing and degenerescence [40]*.* Fibrin crosslinking by factor XIII is of crucial importance not only for hemostasis, but also for inflammation. For instance, factor XIII A-subunit genotype was shown to influence C-reactive protein levels during inflammation in rheumatoid arthritis (RA) [41].

Finally, *SELL* encodes the lymphocyte homing receptor L-selectin/CD62L, one of the major adhesion molecules, which regulates entry of neutrophils and monocytes into inflamed tissues and contributes to the severity of joint inflammation in experimental arthritis [42]. Thus, upregulation of *SELL*, as shown here in DCs could well participate in joint inflammation in SpA. Interestingly, this gene was also found to be upregulated in splenic DCs from the HLA-B27/human β2-microglobulin transgenic rat [21].

## Conclusions

Results of the present study reveal the defective functional capacity of DCs from SpA patients, as compared to controls. Furthermore, our results demonstrate significant changes in MD-DCs gene expression upon LPS stimulation that may be inherent to SpA patients. Some of them (that is, decreased Wnt signaling) could account for the altered DC function that provided a rationale for the present study. Others (that is, upregulation of *ADAMTS15*, *F13A1* and *SELL*) would concur to reinforce tissue inflammation and/or damage. Finally, the co-expression of *CITED2* co-transcriptional factor with several of the foregoing genes (that is, the Wnt signaling pathway and *ADAMTS15*) supports the hypothesis that a coordinated deregulation taking place in DCs may play an important role in SpA pathogenesis.

## Additional files

Additional file 1: Table S1. Characteristics of the study patients.*SpA, spondyloarthritis; BASDAI, Bath Ankylosing Spondylitis Disease Activity Index; NSAID, non-steroidal anti-inflammatory drug; TNF, tumor necrosis factor. ND, not done. *The registered manifestations correspond to those present at the time of examination, or retrieved from past-medical history. **Refers to radiographic sacroiliitis ≥ grade II bilateral or grade III unilateral. ***Six patients are common to both studies. ****Data available for nine patients. Additional file 2: Table S2. Characteristics of the study healthy controls. Additional file 3: Table S3. Nucleotide sequence of the PCR primers. Additional file 4: Figure S1. Comparison of monocyte subsets distribution among peripheral blood mononuclear cells (PBMCs) between spondyloarthritis (SpA) patients and healthy controls (Ctrl) by six-color flow cytometry. Distribution of monocyte subsets among CD45^+^ PBMC was studied in five healthy donors (clear boxes) and 10 HLA-B27^+^ SpA patients (gray boxes). Results are represented as boxes, bars indicate medians. The mean age of healthy donors was 42 years at the time of the study and 40% of them were men. Characteristics of the patients are shown in Additional file 1: Table S1. Additional file 5: Figure S2. Phenotypic characterization of purified monocytes and monocyte-derived dendritic cells (MD-DCs) by flow cytometry. Results of one healthy donor representative of study subjects are shown. Monocytes (D0) were CD14^+^, CD11c^+^, CD40^−^, HLA-I^+^, HLA-DR^+^, CD80^−^, CD83^−^ and CD86^−^. MD-DCs (D7) were CD14^−^, CD11c^+^, CD40^+^, HLA-I^+^, HLA-DR^+^, CD80^−^, CD83^dim^, and CD86^+^. HLA-DR, CD83 and CD86 expression increased, and CD80 was induced on MD-DCs after lipolpolysaccharide (LPS) stimulation for 24 h (H24). Clear plot represents control isotype and gray plot the tested antibody. Additional file 6: Figure S3. Unsupervised hierarchical clustering of 57 samples based on whole-genome gene expression levels. Each time point is represented by a color (baseline (H0): blue, 6 h (H6): red, 24 h (H24): green). Samples are clustered on the horizontal axis (top row: spondyloarthritis (SpA) patients, bottom row: healthy controls). Additional file 7: Table S4. List of the genes differentially expressed in monocyte-derived dendritic cells (MD-DCs) between spondyloarthritis (SpA) and controls, ranked by fold change.**The criteria for the inclusion of genes in this table are described in Patients and Methods. Global values are LInear models for microarray data (LIMMA) values. The four genes selected for qRT-PCR validation are in bold text.

## Abbreviations

AS: ankylosing spondylitis
CPM: counts per minute
DC: dendritic cell
EBV: Epstein-Barr virus
GM-CSF: granulocyte-macrophage colony-stimulating factor
IL: interleukin
LIMMA: LInear models for microarray data
LPS: lipopolysaccharide
mAb: monoclonal antibody
MD-DC: monocyte-derived dendritic cell
MLR: mixed lymphocyte reaction
MMP: metalloproteinase
PBMC: peripheral blood mononuclear cell
qRT-PCR: real-time quantitative PCR
SpA: spondyloarthritis
TNF: tumor necrosis factor
