# Supplementary material for: Monocyte-derived dendritic cells from HLA-B27+ axial spondyloarthritis (SpA) patients display altered functional capacity and deregulated gene expression
Source: Arthritis Res Ther. 2014 Aug 21;16(4):417. doi: 10.1186/s13075-014-0417-0 (PMC4292999; doi:10.1186/s13075-014-0417-0)
Supplement: Additional file 1: Table S1. — Characteristics of the study patients.*SpA, spondyloarthritis; BASDAI, Bath Ankylosing Spondylitis Disease Activity Index; NSAID, non-steroidal anti-inflammatory drug; TNF, tumor necrosis factor. ND, not done. *The registered manifestations correspond to those present at the time of examination, or retrieved from past-medical history. **Refers to radiographic sacroiliitis ≥ grade II bilateral or grade III unilateral. ***Six patients are common to both studies. ****Data available for nine patients. [file 13075_2014_417_MOESM1_ESM.pdf]

Feature	Flow cytometry (n = 10)	Proliferation assay (n = 19)***	Transcriptomic study (n = 9)***
Male gender, n (%)	3 (30)	12 (63)	5 (67)
Age, mean (SD), yrs	43.9 (10.2)	46.4 (9.9)	48.1 (7.9)
HLA-B27, n (%)	10 (100)	18 (94.7)	9 (100)
Disease duration, mean (SD), yrs	23.0 (9.2)	23.2 (10.2)	15.1 (5.2)
BASDAI, mean (SD)	2.9 (1.8)	3.4 (2.2)	3.9 (2.0)
CRP, mean (SD), mg/L	ND	10.6 (14.7)****	7.1 (8.5)
Axial manifestations			
- Inflammatory back pain, n (%)	10 (100)	12 (100)	9 (100)
- Radiographic sacroiliitis**, n (%)	7 (70)	11 (95)	8 (89)
Peripheral manifestations			
- Peripheral arthritis, n (%)	3 (30)	6 (32)	2 (22)
- Enthesitis, n (%)	4 (40)	11 (58)	6 (67)
Extra-articular manifestations			
Uveitis, n (%)	0 (0)	5 (26)	3 (33)
Psoriasis, n (%)	2 (20)	4 (21)	3 (33)
Inflammatory bowel disease, n (%)	0 (0)	2 (10)	0 (0)
Current treatment			
- NSAID, n (%)	3 (30)	14 (74)	5 (56)
- Anti-TNF therapy, n (%)	3 (30)	3 (16)	2 (22)
- None, n (%)	4 (40)	4 (21)	2 (22)
