# Supplementary material for: Monocyte-derived dendritic cells from HLA-B27+ axial spondyloarthritis (SpA) patients display altered functional capacity and deregulated gene expression
Source: Arthritis Res Ther. 2014 Aug 21;16(4):417. doi: 10.1186/s13075-014-0417-0 (PMC4292999; doi:10.1186/s13075-014-0417-0)
Supplement: Additional file 2: Table S2. — Characteristics of the study healthy controls. [file 13075_2014_417_MOESM2_ESM.pdf]

| Feature | Flow cytometry (n = 5) | Proliferation assay (n = 24)*** | Transcriptomic study (n = 10)*** |
|---|---|---|---|
| Male gender, n (%) | 2 (40) | 7 (29.2) | 6 (60) |
| Age, mean (SD), yrs | 44 (16.6) | 44.5 (13.3) | 37.3 (13.3) |
| HLA-B27, n (%) | 0 (0) | 0 (0) | 0 (0) |
