# Supplementary material for: Monocyte-derived dendritic cells from HLA-B27+ axial spondyloarthritis (SpA) patients display altered functional capacity and deregulated gene expression
Source: Arthritis Res Ther. 2014 Aug 21;16(4):417. doi: 10.1186/s13075-014-0417-0 (PMC4292999; doi:10.1186/s13075-014-0417-0)
Supplement: Additional file 3: Table S3. — Nucleotide sequence of the PCR primers. [file 13075_2014_417_MOESM3_ESM.pdf]

| **Gene name** | **Forward primer** | **Reverse primer** |
|---|---|---|
| Candidate genes | | |
| ADAMTS15 | GACTCTTCACCAAGCCCATGC | AGCCAGGTAGTTGTCATCCCC |
| CITED2 | TGGTGATAGAAATGGGTTTGG | GTTTCGATCGAGTCAACAGC |
| F13A1 | GTGAAGATGATGCTGTGTATCTG | ATGCCATCTTCAAACTGACC |
| SELL | CAGTCTACCTGCAGCACAGC | TGGGTGCTCTGACATTTC |
| Housekeeping genes | | |
| ACTB | GGACTTCGAGCAAGAGATGG | AGGAAGGAAGGCTGGAAGAG |
| GAPDH | CCTCAACGACCACTTTGTCA | GAGGGTCTCTCTCTTCCTCTTGT |
| RPL30 | CCTAAGGCAGGAAGATGGTG | AATGACCAATTTCGCTTTGC |
