# Supplementary figures and images for: Monocyte-derived dendritic cells from HLA-B27+ axial spondyloarthritis (SpA) patients display altered functional capacity and deregulated gene expression
Source: Arthritis Res Ther. 2014 Aug 21;16(4):417. doi: 10.1186/s13075-014-0417-0 (PMC4292999; doi:10.1186/s13075-014-0417-0)

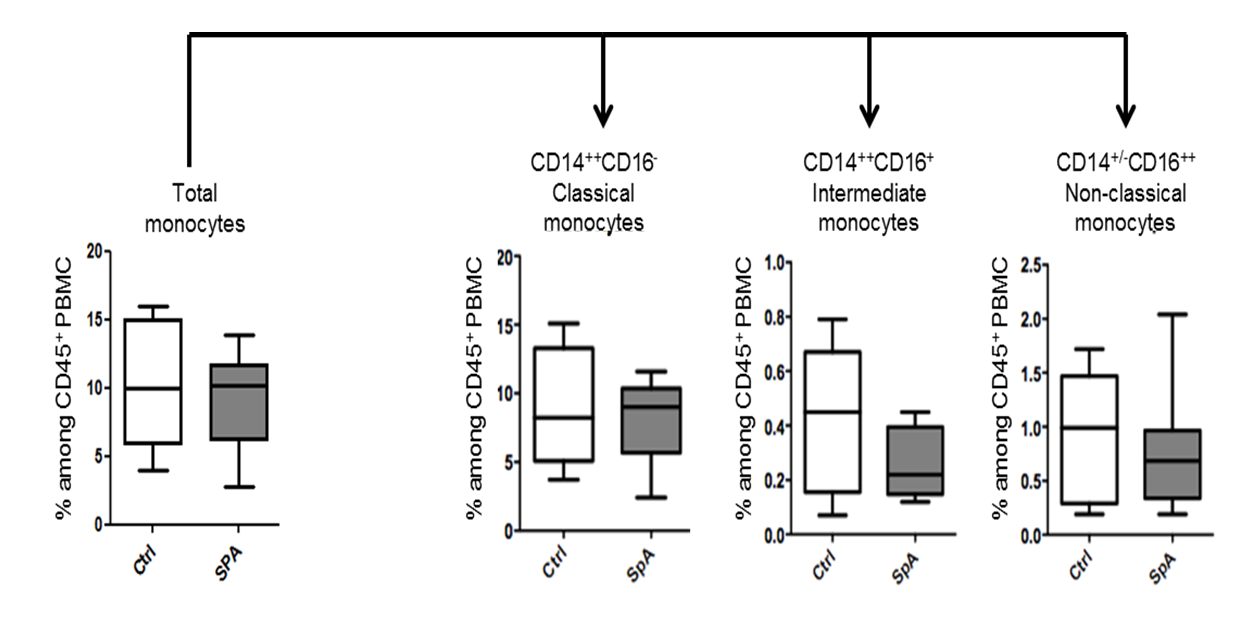

Supplement: Additional file 4: Figure S1. — Comparison of monocyte subsets distribution among peripheral blood mononuclear cells (PBMCs) between spondyloarthritis (SpA) patients and healthy controls (Ctrl) by six-color flow cytometry. Distribution of monocyte subsets among CD45+ PBMC was studied in five healthy donors (clear boxes) and 10 HLA-B27+ SpA patients (gray boxes). Results are represented as boxes, bars indicate medians. The mean age of healthy donors was 42 years at the time of the study and 40% of them were men. Characteristics of the patients are shown in Additional file 1: Table S1. [file 13075_2014_417_MOESM4_ESM.tiff]

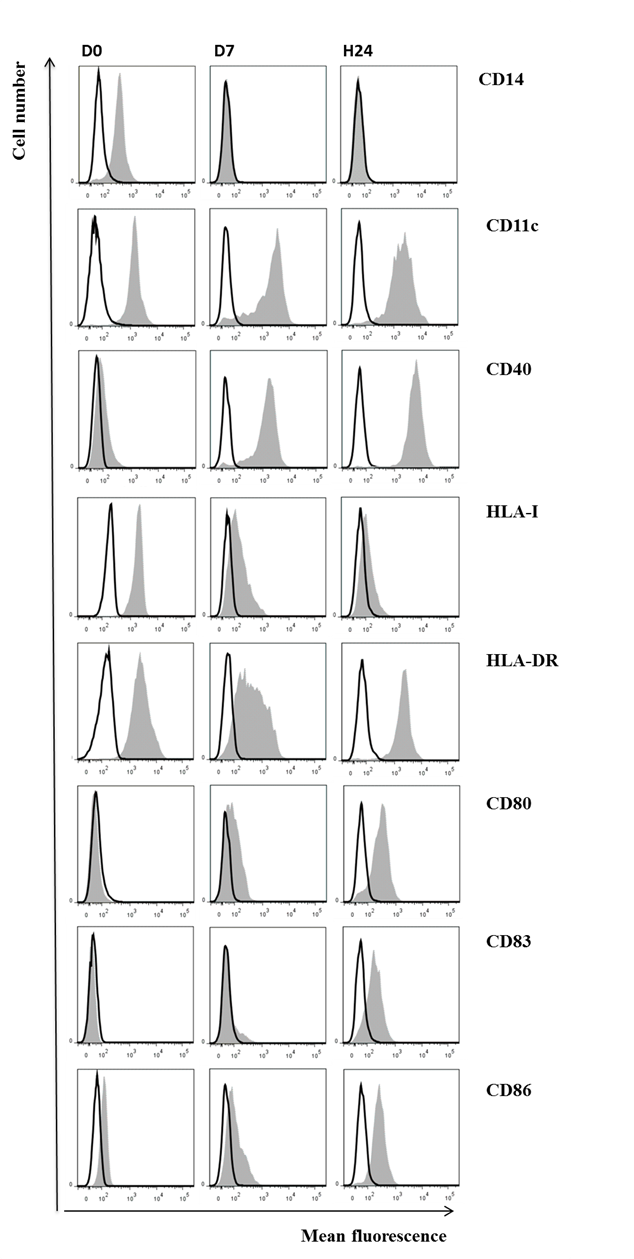

Supplement: Additional file 5: Figure S2. — Phenotypic characterization of purified monocytes and monocyte-derived dendritic cells (MD-DCs) by flow cytometry. Results of one healthy donor representative of study subjects are shown. Monocytes (D0) were CD14+, CD11c+, CD40−, HLA-I+, HLA-DR+, CD80−, CD83− and CD86−. MD-DCs (D7) were CD14−, CD11c+, CD40+, HLA-I+, HLA-DR+, CD80−, CD83dim, and CD86+. HLA-DR, CD83 and CD86 expression increased, and CD80 was induced on MD-DCs after lipolpolysaccharide (LPS) stimulation for 24 h (H24). Clear plot represents control isotype and gray plot the tested antibody. [file 13075_2014_417_MOESM5_ESM.tiff]

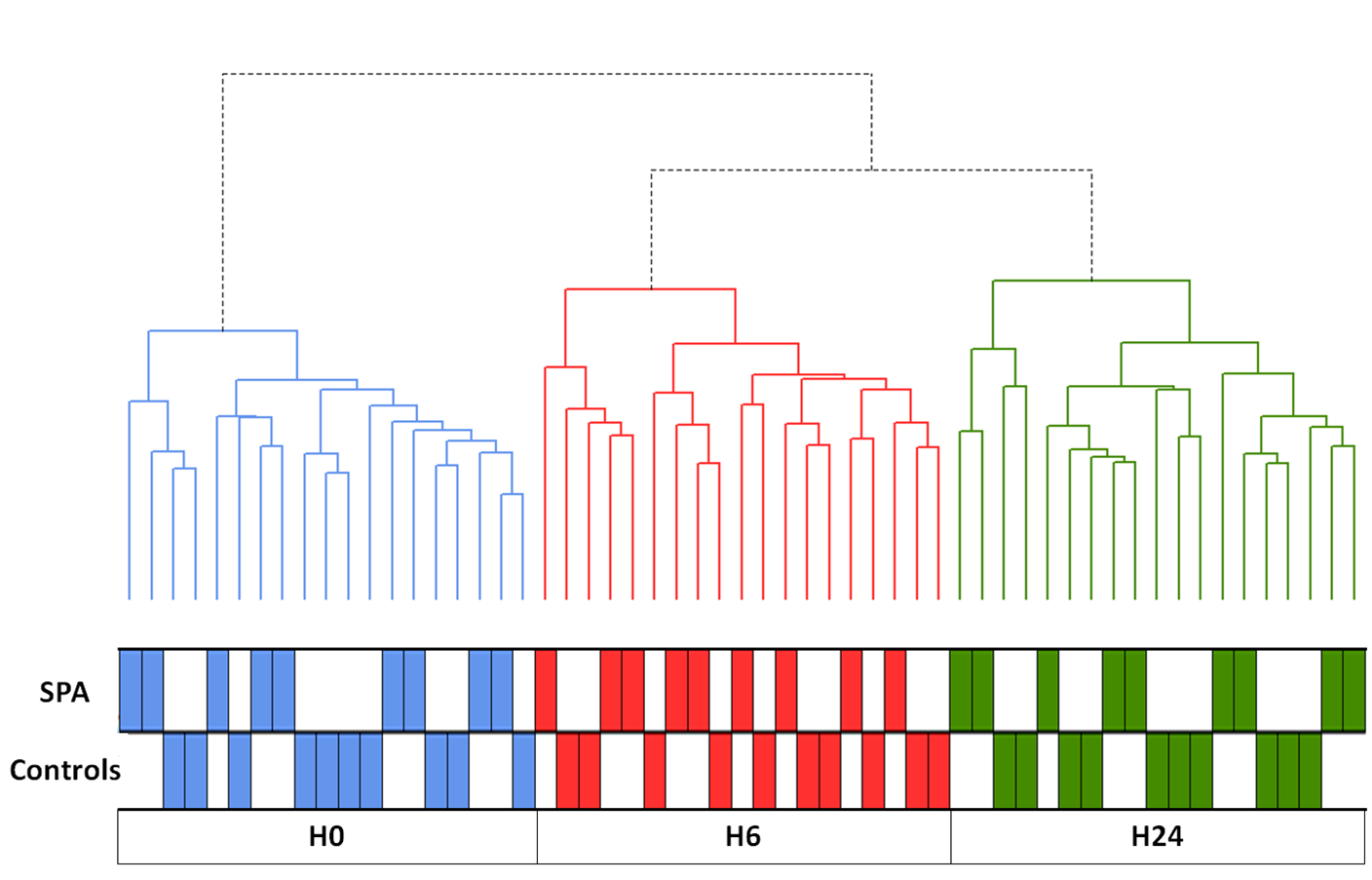

Supplement: Additional file 6: Figure S3. — Unsupervised hierarchical clustering of 57 samples based on whole-genome gene expression levels. Each time point is represented by a color (baseline (H0): blue, 6 h (H6): red, 24 h (H24): green). Samples are clustered on the horizontal axis (top row: spondyloarthritis (SpA) patients, bottom row: healthy controls). [file 13075_2014_417_MOESM6_ESM.tiff]
