# Supplementary material for: Monocyte-derived dendritic cells from HLA-B27+ axial spondyloarthritis (SpA) patients display altered functional capacity and deregulated gene expression
Source: Arthritis Res Ther. 2014 Aug 21;16(4):417. doi: 10.1186/s13075-014-0417-0 (PMC4292999; doi:10.1186/s13075-014-0417-0)
Supplement: Additional file 7: Table S4. — List of the genes differentially expressed in monocyte-derived dendritic cells (MD-DCs) between spondyloarthritis (SpA) and controls, ranked by fold change.**The criteria for the inclusion of genes in this table are described in Patients and Methods. Global values are LInear models for microarray data (LIMMA) values. The four genes selected for qRT-PCR validation are in bold text. [file 13075_2014_417_MOESM7_ESM.pdf]

Gene name	Ensembl ID	P-Value	Global Fold change
Up-regulated genes			
TRBC2	ENSG00000211772	9.67E-03	2.96
**SELL**	**ENSG00000188404**	**6.06E-03**	**2.96**
**F13A1**	**ENSG00000124491**	**6.12E-05**	**2.63**
**ADAMTS15**	**ENSG00000166106**	**3.02E-04**	**2.38**
RND3	ENSG00000115963	8.84E-03	2.36
SIGLEC15	ENSG00000197046	7.98E-04	2.12
HSPA1A	ENSG00000204389	6.03E-03	2.01
TBCK	ENSG00000145348	1.05E-03	1.99
BACE2	ENSG00000182240	8.46E-03	1.85
FBXL4	ENSG00000112234	5.74E-03	1.85
PIAS2	ENSG00000078043	5.35E-03	1.82
KIAA0907	ENSG00000132680	2.26E-03	1.78
SEMA3C	ENSG00000075223	7.04E-03	1.74
HSPH1	ENSG00000120694	5.82E-03	1.74
EDEM3	ENSG00000116406	5.89E-03	1.71
USP40	ENSG00000085982	8.55E-03	1.65
P4HA1	ENSG00000122884	6.44E-03	1.64
DNAJA4	ENSG00000140403	1.92E-03	1.63
FBXO18	ENSG00000134452	2.96E-03	1.54
PTPLA	ENSG00000165996	5.83E-03	1.52
Down-regulated genes			
NDP	ENSG00000124479	3.21E-03	0.13
OLR1	ENSG00000173391	3.36E-03	0.14
FAIM2	ENSG00000135472	6.58E-04	0.14
ZNF804A	ENSG00000170396	8.11E-03	0.23
PLP2	ENSG00000102007	3.42E-03	0.29
CSF3R	ENSG00000119535	5.64E-04	0.32
EIF4H	ENSG00000106682	1.11E-04	0.34
BCKDHA	ENSG00000248098	2.30E-03	0.35
SGMS2	ENSG00000164023	4.13E-05	0.36
GTSF1	ENSG00000170627	6.81E-03	0.36
**CITED2**	**ENSG00000164442**	**7.29E-04**	**0.40**
COX7B	ENSG00000131174	5.87E-03	0.40
LRRC4	ENSG00000128594	8.03E-05	0.42
MNDA	ENSG00000163563	3.75E-03	0.44
ANKRD36BP1	ENSG00000214262	8.41E-03	0.44
HAUS1	ENSG00000152240	9.11E-04	0.44
TSPYL5	ENSG00000180543	4.77E-04	0.45
RPL26	ENSG00000161970	3.94E-03	0.46
PIGB	ENSG00000069943	1.58E-03	0.47
RPS15AP1	ENSG00000214535	4.65E-03	0.48
CKAP2	ENSG00000136108	6.45E-03	0.50

ALG10B	ENSG00000175548	4.90E-03	0.51
RBBP9	ENSG00000089050	2.01E-03	0.51
PARVG	ENSG00000138964	9.96E-03	0.52
P2RX1	ENSG00000108405	8.45E-06	0.52
RPL10A	ENSG00000198755	7.17E-03	0.53
SPATA20	ENSG00000006282	7.41E-03	0.53
GPR180	ENSG00000152749	9.34E-03	0.54
TTC39C	ENSG00000168234	2.79E-04	0.54
RPS4X	ENSG00000198034	6.85E-03	0.54
ENY2	ENSG00000120533	5.10E-03	0.54
ANAPC15	ENSG00000110200	1.39E-03	0.55
FHL3	ENSG00000183386	7.01E-03	0.55
WDR25	ENSG00000176473	4.45E-05	0.56
FAU	ENSG00000149806	6.03E-04	0.56
TRIM24	ENSG00000122779	1.29E-03	0.57
RFC3	ENSG00000133119	1.03E-03	0.57
TMEM205	ENSG00000105518	4.43E-04	0.57
ITPRIP	ENSG00000148841	7.55E-03	0.58
PORCN	ENSG00000102312	8.47E-03	0.58
BLOC1S1	ENSG00000135441	3.99E-03	0.58
CRTAP	ENSG00000170275	4.34E-03	0.59
ELMO1	ENSG00000155849	6.58E-03	0.59
NUDT3	ENSG00000112664	4.60E-06	0.60
USP30	ENSG00000135093	3.20E-03	0.60
COX20	ENSG00000203667	4.08E-03	0.61
CTBP1-AS1	ENSG00000196810	5.98E-03	0.61
TNFSF13B	ENSG00000102524	3.15E-04	0.61
IFT52	ENSG00000101052	3.79E-03	0.61
RPS15	ENSG00000115268	4.69E-03	0.62
ACADM	ENSG00000117054	9.23E-03	0.62
TFAM	ENSG00000108064	6.40E-04	0.63
MBIP	ENSG00000151332	8.04E-04	0.63
POU5F1B	ENSG00000212993	1.55E-04	0.63
MUT	ENSG00000146085	2.72E-03	0.63
SLU7	ENSG00000164609	1.50E-04	0.63
BAK1P1	ENSG00000175730	4.24E-03	0.64
ZFP36L2	ENSG00000152518	9.55E-03	0.65
SAP130	ENSG00000136715	1.15E-04	0.65
POLR1D	ENSG00000186184	2.24E-04	0.65
FAM204A	ENSG00000165669	7.35E-05	0.66
